# Epidemiology, microbiological and clinical characteristics of Enterococcus species bloodstream infections: A 10-year retrospective cohort study from Qatar

**DOI:** 10.1016/j.amsu.2022.104258

**Published:** 2022-08-03

**Authors:** Gawahir A. Ali, Wael Goravey, Mostafa Suhail Najim, Khalid M. Shunnar, Shahd I. Ibrahim, Joanne Daghfal, Emad B. Ibrahim, Muna Al Maslamani, Ali S. Omrani, Hamad Abdel Hadi

**Affiliations:** a Department of Infectious Diseases, Communicable Diseases Centre, Hamad Medical Corporation, Doha, Qatar; b Department of Internal Medicine, Hamad Medical Corporation, Doha, Qatar; c Department of Laboratory Medicine and Pathology, HMC, Doha and Qatar University, Biomedical Research Centre, Doha, Qatar

**Keywords:** *Enterococcus*, Bacteremia, Blood stream infection (BSI), Qatar

## Abstract

**Background and objective:**

Enterococcus species is one of the leading causes of community and healthcare-associated infections resulting in significant morbidity and mortality. In this study, we aim to evaluate the epidemiology, microbiological and clinical characteristics of Enterococcus Blood Stream Infections (BSIs) over 10 years period in a national secondary care setting.

**Methods:**

A retrospective cohort study was conducted on verified cases of *enterococcal* BSIs in adults from January 2009–December 2018 from specialized care hospitals at Hamad Medical Corporation, Qatar. Epidemiological, microbiological and clinical data were reported and analyzed.

**Results:**

A total of 263 *enterococcus* BSIs cases were identified, predominant were males (65%) with a median age of 63 (IQR 48–74). *E. faecalis* and E. faecium were predominate at 93.5% (73.38% and 20.15% respectively). Diabetes was the commonest premorbid condition (54.3%) followed by chronic kidney disease (36.5%). Central lines and genitourinary were the most common sources (18.25%, 14.83% respectively) while no identified source was reported in 45.25% of cases. Ampicillin susceptibility was 82.51% while vancomycin resistance was reported in 10.6% of isolates. Successful bacteremia clearance was achieved in 81.37% of cases at a mean of 4 days (Range 2–5 days) while metastatic complications occurred in 5.3% of cases. Univariate mortality risk analysis was associated with ICU admission, low level of consciousness, high bacteremia scores, and presence of catheters. The 30 days mortality was high at 66.54% with CKD and cancer patients at the highest mortality risks (OR 16.334 (CI 4.2–62.4) and 16 (CI 3–84) respectively.

**Conclusion:**

Significant mortality was associated with *enterococcus* BSI despite low rates for ampicillin and vancomycin resistance necessitating early identification of susceptible patients to instigate suitable preventive measures.

## Highlights

• Enterococcus bacteremia cases were enrolled retrospectively over a decade timeframe.
• *E. faecalis* and E. faecium constitute the majority at 93.5%.
• Significant mortality was associated with *enterococcus* bloodstream infection.
• Low rates for ampicillin and vancomycin resistance.
• Enterococcus bacteremia carries high mortality in CKD and cancer patients.

## 1 Introduction

Human *enterococcus* species are gram-positive gastrointestinal commensals with low pathogenicity but capable of causing invasive diseases with significant morbidity and mortality particularly in hospitalized patients with premorbid conditions, invasive procedures, and the immune-compromised [1]. Of the pathogenic disease spectrum, the two main species, *Enterococcus faecalis* (*E. faecalis*) and *Enterococcus faecium* (E. faecium) account for most of the clinical pathology with the former being widely associated with the community while the latter with Healthcare-Associated Infections (HCAIs) [2].

In contrast to other susceptible gram-positive cocci, *enterococci* possess unique innate and acquired resistance mechanisms resulting in distinctive cell wall characteristics that generate diverse resistance towards commonly directed antimicrobials including cephalosporins, aminoglycosides, and glycopeptides [3]. Over the past decades, enterococcal antimicrobial resistance has been escalating in many regions around the world leading to the ominous outcomes of HCAIs secondary to Vancomycin-Resistant *Enterococci* (VRE) with high morbidity and mortality, substantial challenges in control, prevention, and management including economic consequences [4,5]. More recently, with the advances in modern medical technologies, invasive interventions in patients with a multitude of premorbid conditions as well as the predominance of immune-suppressive states, the global prevalence of enterococcus Blood Stream Infections (BSIs) has been increasingly reported [2,6]. The problem including VREs has regional variations regarding epidemiology, microbiological characteristics as well as clinical outcomes [7,8]. This variability has been reflected in prevalence diversity, variations in resistance profiles with reported VREs as high as 60% in some regions while crude mortality rates range from 21.4% (low estimates) to 64.2% (high estimates) depending on local settings and patients’ demographics [5,6,9]. The reported risk factors for developing *enterococcal* BSIs are advanced age, prolonged hospital stay, surgical and invasive procedures, admission to critical care, comorbidities including immunosuppression and organ transplantation as well as neoplastic diseases [10]. In addition to the mortality of the acute infection, a wide range of complications has been reported including healthcare-associated pneumonia, infective endocarditis, meningitis, soft tissues, and bone infections [2].

In Qatar, local reports and observations highlighted the frequent occurrence of *enterococcal* BSIs including occasional VREs which were not supported by accurate local or regional data. This study aimed to evaluate the epidemiology, examine risk factors for acquisition, assess microbiological characteristics, and describe clinical outcomes of *enterococcal* BSIs in Qatar over 10-years. Understanding the spectrum of this important community and hospital-acquired pathogen will assist in comprehending the scale of the problem, developing future control and prevention programs as well as ultimately improving clinical outcomes.

## 2 Methods

We performed a retrospective cohort study of adult patients who developed *enterococcal* BSIs over 10 year period from 1st of January 2009–31st of December 2018 collected from acute care hospitals as well as specialized secondary care settings at 10.13039/100007833Hamad Medical Corporation in Qatar which comprises acute medical and surgical admissions supported by medical and surgical critical care units in addition to specialized ambulatory, heart, women, cancer, rehabilitation, and long-term facilities with total beds capacity of 2491. The study was registered in the Research Registry database (https://www.researchregistry.com/) with unique number: **researchregistry8031**. All methods were performed in accordance with the relevant guidelines and regulations by Hamad Medical Corporation's Institutional Review Board (MRC) and the declaration of Helsinki. The research was retrospectively registered and has been reported in line with the STROCSS criteria [11]. All patients aged ≥14 years with at least one clinically relevant positive blood culture for *Enterococcus* species were eligible for inclusion in the study following fulfilling study definitions. Qatar is one of the Gulf countries in the Arabian Peninsula with a dynamic and diverse population that grew substantially over the last two decades. Estimates of the population in 2009 was 1.8 million which grew steadily to 2.8 million in 2019.

### 2.1 Study definitions

BSI was defined as at least one positive blood culture for one of the Enterococcus species with clinical signs and/or findings of infection. In patients with persistent BSIs caused by the same organism, only the first episode was included. If patients had more than one episode or more separate BSIs within the same study period, only one infection will be considered for the analysis to reduce associated stratifying risk factors, but the analysis were included for microbiological tests for accumulated resistance as well as clinical outcomes. The source of the BSI was determined using bacteriologic sampling at the presumed source and medical reports of the clinical examination and assessment. A second infectious disease physician was consulted for controversial cases. The presence of two or more clinically important organisms isolated from a single blood culture sample defines polymicrobial BSIs. Appropriate antimicrobial therapy was defined as active antimicrobial against Enterococcus species with adequate dosage within 5 days from the onset whereas empirical antibiotic therapy was initiated within 24 h after the onset of BSIs and before the report of antibiotic susceptibility results.

### 2.2 Data collection

Following identification of cases, patients' electronic as well as paper format medical and laboratory records were reviewed. Data were collected including demographics, microbiological and clinical characteristics as well as management and clinical outcomes. Out of 309 cases identified, 263 were included after fulfilling study inclusion criteria. All identified cases were included in the final analysis.

### 2.3 Microbiological identification and antimicrobial susceptibility testing (ID/AST)

The retrospective study was conducted on routine processed blood cultures samples received by the Microbiology Division, Department of Laboratory Medicine and Pathology, HMC, Qatar between 1st of January 2009- 1st to December 31, 2018. All identified bacterial isolates were collected from general and specialist hospitals serviced by HMC. Microbiological identifications and AST were performed using standard microbiological techniques that are regularly inspected and approved by the Clinical and Laboratory Standards Institute (CLSI) [12]. Throughout the study, bacterial isolates were identified using the 10.13039/100017412BD Phoenix™ Automated Microbiology System while Matrix-Assisted Laser Desorption Ionization-Time of Flight Mass Spectrometry (MALDI-10.13039/100001780TOF MS) using the Bruker Daltonics MALDI Biotyper (Billerica, MA, USA), according to the manufacturer's instructions was used for supporting identification following its introduction at the facility in 2015. All antibiotics susceptibility testing was performed by BD Phoenix using the NMIC/ID-94 panel according to the manufacturer's recommendations (BD Biosciences, Heidelberg, Germany) and the minimum inhibitory concentration (MIC) values were interpreted following the timely recommendations from the CLSI.

### 2.4 Statistical analysis

Data were analyzed using STATA software (Stator., College Station, TX, USA). Categorical variables were expressed as frequency counts and percentages with a 95% confidence interval. Continuous variables were expressed as medians. To identify risk factors associated with mortality, a multivariate logistic regression model with a backward method was used to control the effects of confounding variables. Statistically significant variables (p < 0.1) to mortality in the univariate logistic analyses were chosen to build a multivariate model to compute odds ratios (ORs). Throughout data analysis, statistical significance was set at p < 0.05.

## 3 Results

### 3.1 Demographic and clinical characteristics

Over the 10 years study period, a total of 263 patients developed *enterococcal* BSIs with male predominance (65.02%, n = 171) and a median age of 63 years (IQR 48–74) as depicted in Table 1. Diabetes mellitus was the most common premorbid condition at (54.37%, n = 143) followed by chronic kidney diseases (36.50%, n = 96). Charlson Comorbidity Index (CCS) median score was 4 (range 2–7) while Pitt Bacteremia Score (PBS) median was 3 (0–8) for the cohort. Most episodes occurred at critical care units (CCU) (59.70%, n = 157) with one-third of cases underwent substantial surgical procedures (31.18%, n = 82) within 3 months before the onset of bacteremia. Urinary catheterization and central lines were risk factors in (62.74%, n = 165) and (50.95%, n = 134) patients reflecting critical care. The commonest evaluated sources of BSIs were Central line-associated Bloodstream Infections (CLABSIs) (18.25%, n = 48) followed by genitourinary tract (GUT) (14.83%, n = 39) whereas no identified source was reported in almost half of the cases (45.25%, n = 119). Table 1.

**Table 1:** Clinical characteristics of patients with enterococcal BSIs.

Variable	Total (N = 263)
Male sex	171 (65.02%)
Age	63.00 (48.00–74.00)
Nationality by WHO region of origin	
Eastern Mediterranean Region	216 (82.13%)
South-East Asia Region	37 (14.07%)
Western Pacific Region	7 (2.66%)
European Region	2 (0.76%)
African Region	1 (0.38%)
Co-existing medical condition	
Diabetes mellitus	143 (54.37%)
Chronic kidney diseases	96 (36.50%)
Cardiovascular diseases	83 (31.56%)
Cerebrovascular diseases	76 (28.90%)
Active malignancy	62 (23.57%)
Dementia	37 (14.07%)
Chronic liver diseases	30 (11.41%)
Chronic lung disease	28 (10.65%)
Connective tissue disease	3 (1.14%)
Charlson Comorbidity Index (CCS)	4.00 (2.00–7.00)
Pitt Bacteremia Score	3.00 (0.00–8.00)
Hypotension	99 (37.64%)
Cardiac Arrest	42 (15.97%)
Mental Status	
Alert	118 (44.87%
Comatose	92 (34.98%)
Disoriented	36 (13.69%)
Stuporous	17 (6.46%)
ICU admission	157 (59.70%)
Surgical procedures	82 (31.18%)
Urinary catheterization	165 (62.74%)
Mechanical ventilation	104 (39.54%)
Central line insertion	134 (50.95%)
Immune Suppression	6 (2.28%)
Source	
Uncertain	119 (45.25%)
CLABSI	48 (18.25%)
GUT	39 (14.83%)
SST	20 (7.60%)
GIT	17 (6.46%)
IE	9 (3.42%)
BJI	6 (2.28%)
RT	4 (1.52%)
CNS	1 (0.38%)

*Data are presented as number (percentage) or median (interquartile range); WHO, World Health Organization; ICU, intensive care unit; CLABSI, central line bloodstream infection; GUT, Genitourinary tract; SSI, Skin and Soft tissue; GIT, Gastrointestinal tract; IE, Infective Endocarditis; BJI, Bone and Joint Infections; RT, Reproductive Rract; CNS, Central Nervous System.

### 3.2 Microbiological characteristics

The most common *Enterococcus* species was E. *faecalis* comprising 73.38%, (173/263) followed by E. *faecium* 20.15%, (53/263) with a combined prevalence of 93.5%. Concomitant bacteremia with other pathogens included E. *coli* 28 (10.65%) followed by *Klebsiella* species in 5.70% (15). Among the 263 episodes of all species BSIs, Ampicillin was sensitive in 82.51% (217/273) while vancomycin resistance (VRE) was observed in only 10.6% (28/263), Table 2.

**Table 2:** Microbiological characteristics of patients with enterococcal BSIs.

Enterococcus species	
*E. faecalis*	193 (73.38%)
E. faecium	53 (20.15%)
Other species	17 (6.46%)
Polymicrobial bacteremia	
Second microorganism	
*E. coli*	28 (10.65%)
Klebsiella sp	15 (5.70%)
Psuedomonas sp	9 (3.42%)
Enterobacter sp	8 (3.04%)
Candida sp	3 (1.14%)
Antimicrobials sensitivity	
Ampicillin S	217 (82.51%)
Ciprofloxacin S	65 (24.71%)
Daptomycin S	190 (72.24%)
Gentamycin S	166 (63.12%)
Linezolid S	211 (80.23%)
Vancomycin S	235 (89.35%)

*Data are presented as number (percentage) or median (interquartile range); S, Sensitivity.

### 3.3 Outcomes

The bacteremia was cleared in most cases (81.37%, n = 214) at a mean of 4 days (2–5), while mean hospital stay days before the onset of bacteremia was reported as 10 (1–23). Metastatic infective complications were rare following primary episodes of bacteremia (5.3%, n = 14) with infective endocarditis constituting 3.4% (n = 9) while the mean duration of hospital stay following acquisition was 16 days (7−24). Despite the use of appropriate antibiotics in most patients (95.06%, n = 250), the 30 days mortality was high at 66.54% (n = 175) Table 3. Univariate analyses for mortality risk were associated with a low level of consciousness at the time of bacteremia, Pitts Bacteremia Score, ICU admission, urinary and central intravenous catheters (Table 4). Patients with CKD and Cancer showed a higher risk of 30-day all-cause mortality with an adjusted odds ratio of 16.334 (CI 4.278–62.358) and 16.030 (CI 3.056–84.078) respectively. Also, the presence of urinary catheterization was associated with risk factor for mortality (OR 4.9, 95% CI 1.26–19) (Table 4).

**Table 3:** Outcomes of patients with enterococcal BSIs.

Complications	1 (0.38%)
Abscess	1 (0.38%)
Infective endocarditis	1 (0.38%)
Septic Arthritis intra-abdominal collection	2 (0.76%)
osteomyelitis	1 (0.38%)
No complication	249 (94.68%)
Clinical outcomes	
Length of hospital stay _preBSI (days)	10.00 (1.00–23.00)
Length of hospital stay in days (Post diagnosis)	16.00 (7.00–24.00)
Clearance of bacteremia	214 (81.37%)
Clearance of bacteremia (days)	4.00 (2.00–5.00)
Appropriate empirical Antibiotics	250 (95.06%)
Mortality by day 30	175 (66.54%)
Mortality by day 90	244 (92.78%)

*Data are presented as number (percentage) or median (interquartile range).

**Table 4:** Logistic regression for 30-day all-cause mortality.

Univariate logistic regression			Multi-variate logistic regression			
Variable	Unadjusted odds ratio	95% Confidence interval	P-Value	Adjusted odds ratio	95% Confidence interval	P-Value
Age	1.014	1.000–1.029	0.057			
Charlson comorbidity index	1.205	1.114–1.303	0.000	0 .997	0 .815–1.221	0.983
Pitt Bacteremia Score	1.316	1.222–1.417	0.000	2.397	0.897–6.403	0.081
Diabetes	1.243	0.741–2.085	0.409			
Chronic kidney disease	2.045	1.207–3.465	0.008	16.334	4.278–62.358	0.000
Cancer	3.619	2.002–6.543	0.000	16.030	3.056–84.078	0.001
Chronic liver disease	2.555	1.184–5.515	0.017	3.396	0.441–26.111	0.240
Hypotension	4.246	2.465–7.313	0.000	0 .057	0 .004–0.754	0.030
Mechanical ventilation	3.681	2.151–6.300	0.000	0 .141	0 .009–2.066	0.153
Cardiac arrest	19.5	7.783–48.855	0.000	0 .572	0 .006–48.181	0.805
Urinary catheter	2.734	1.529–4.887	0.001	4.908	1.263–19.067	0.022
Surgery	0.445	0.244–0.814	0.009	1.427	0.446–4.562	0.549
Intensive care unit admission	3.285	1.839–5.867	0.000	2.376	0.609–9.268	0.212
Central lines	2.924	1.705–5.016	0.000	1.413	0.390–5.118	0.598
Immunosuppression	0.390	0.044–3.397	0.394	–	–	–
Length of hospital stay pre-BSI	1.002	0.996–1.009	0.343	–	–	–
**Source**						
BJI	1.000	–	–	1.000	–	–
CLABSI	0.788	0.398–1.559	0.495	0.301	0.076–1.186	0.086
CNS	1.000	–	–	1.000	–	–
GIT	0.370	0.114–1.202	0.098	0.167	0.017–1.622	0.123
GUT	0.263	0.107–0.643	0.003	0.246	0.044–1.350	0.107
IE	1.000	–	–	1.000	–	–
RT	1.203	0.164–8.831	0.855	0.815	0.060–11.018	0.878
SST	0.133	0.029–0.602	0.009	0.0412	0.0003–5.925	0.214
Uncertain	1.000	–	–	1.000	–	–
**Species**						
single	3.107	0.368–26.218	0.297	–	–	–
*E. faecalis*	0.258	0.042–1.593	0.145	–	–	–
E. faecium	0.869	0.134–5.641	0.884	–	–	–
Mixed	1.000	–	–	–	–	–
days_to_clearance of bacteremia	1.053	0.910–1.218	0.486	–	–	–
**Antimicrobials**						
Amp						
S	0.260	0.133–0.507	0.000	0.180	0.042–0.767	0.020
**Cip**						
R	1.655	0.778–3.523	0.190	–	–	–
S	1.067	0.454–2.507	0.882	–	–	–
**Dap**						
S	0.780	0.266–2.286	0.651	–	–	–
**Gn**						
S	0.714	0.420–1.214	0.214	–	–	–
**Liz**						
R	0.700	0.064–7.602	0.769	–	–	–
S	1.087	0.486–2.433	0.838	–	–	–
**Van**						
S	0.260	0.084–0.803	0.019	0.082	0.006–1.000	0.050
Appropriate empirical antimicrobials	1.138	0.340–3.805	0.833	–	–	–
Length of hospital stay post diagnosis	0.902	0.872–0.934	0.000	–	–	–
**Complications**						
Abscess	1.000	–	–	–	–	–
IE	1.000	–	–	–	–	–
No	0.527	0.032–8.539	0.653	–	–	–
Separate intra abdominal collection from septic arthritis	1.000	–	–	–	–	–
Osteomyelitis	1.000	–	–	–	–	–
	1.000	–	–	–	–	–

*CLABSI, central line bloodstream infection; GUT, Genitourinary tract; SSI, Skin and Soft tissue; GIT, Gastrointestinal tract; IE, Infective Endocarditis; BJI, Bone and Joint Infections; RT, Reproductive Tract; CNS, Central Nervous System; S, Sensitivity; R, Resistant.

## 4 Discussion

The large regional study over a decade timeframe recorded all episodes of enterococcal BSI from acute and specialized hospitals from the main healthcare provider hospitals in the country that almost represent the national profile including prevalence, microbiological and clinical characteristics as well as outcomes. In concordance with other regions, E. *faecalis* and E. *faecium* constitute the majority of enterococcus BSI at 93.5% [7]. Older male patients with comorbidities particularly diabetes and chronic kidney disease (CKD) at higher risks of acquisitions and patients with CKD and cancer have higher attributed mortality while in contrast to other studies, age or gender alone were not associated with increased mortality [13,14]. From the study, it is imperative to highlight that, most of the episodes of bacteremia were isolated from critical care units (CCUs) in 59.6% of cases who had invasive devices such as central lines and urinary catheters. In healthcare settings, It is axiomatic that patients at CCUs are at utmost vulnerability for consequential critical care complications including HCAIs such as secondary bacteremia because of preexisting comorbidities, invasive devices, colonization, and prevalence of resistant organisms in the surrounding environment as well as frequent breach of natural protective barriers [[15], [16], [17]]. These observations have been reciprocated in the study, where most cases were isolated from CCUs and central and urinary catheters were the foremost routes of acquisition. Such observations emphasize the importance of continuously establishing pillars of infection control and prevention measures at CCUs to prevent associated infections [18].

In concordance with previous bacteremia studies, the relevance of the Pitt Bacteremia Score (PBS) was associated with increased morality since it has been validated for both gram-positive and gram-negative bacterial infections including hazardous outcomes in patients with enterococcal BSIs [19,20]. Of note, almost one-third of cases of the cohort underwent surgical procedures within the preceding three months recognized as an acquisition risk factor. Surgical procedures particularly, particularly of the gastrointestinal tracts, are associated with frequent BSIs including enterococci either as a consequence of direct invasion, secondary to surgical site infections in addition to translocation particularly in critically ill patients [21,22]. In addition, although the source of enterococcus BSI was identified in a sizable proportion of our cohort, in contrast to other studies, no obvious source of bacteremia has been reported in the majority of our patients [2,10]. This could be partially attributed to the strict application of the definitions for CLABSI or other HCAIs by the local infection control and prevention teams. Distinctively we report lower rates for ampicillin and vancomycin resistance unlike neighboring and regional countries as well as the global enterococcal resistance particularly for VRE [7,23]. This intriguing result examined against the high usage of vancomycin as the main glycopeptide in our institutions might be explained by the lower rates of prevalence and colonization of VRE, absence of endemic clones, or host factors that might be explored in further future studies. Reported susceptibility profiles are reflected at the high proportion of patients receiving appropriate therapy (95%), with associated bacteriological clearance and low rates of metastatic complications albeit was not sufficient to reduce the high 30 days mortality (66%). Of related complications, the prevalence of infective endocarditis is rare (3.4%, 9 cases) amongst the cohort although echocardiogram is not routinely used in our institution to screen for secondary IE in enterococcal BSIs particularly when other obvious sources have been identified. This is certainly a contentious area where a scoring system such as NOVA and DENOVA has been advocated to aid risks stratification to guide selective echocardiography during management [24,25]. On the other hand, examined against our observations, many studies demonstrated reduction of mortality with appropriate empirical antimicrobial therapy [26,27]. That might not be possible to examine in our cohort since less than 5% of cases received inadequate therapy. Despite that we emphasize the high observed attributed mortality associated with enterococcal BSI is worth highlighting since it is in line with other similar higher estimates observational studies [2,8,19]. Nevertheless, we acknowledge this might be secondary to other confounding factors such as existing main pathology, premorbid conditions, or alternative complications. Additionally, in agreement with other related studies, higher risks of adjusted 30-day all-cause mortality were shown to be high in patients with CKD and cancer that merits careful attention when encountered in the context of enterococcus BSI [14]. Conversely, mortality risk factors were associated with low levels of consciousness (coma and stuporous), Pitt Bacteremia Score, ICU admission, central lines, and urinary catheterization all associated with serious conditions and the need for critical care monitoring. Remarkably, we did not observe any differences in mortality with polymicrobial bacteremia, specific enterococcal species, source identification, or length of hospital stay unlike other similar studies [2,14,28]. While examining microbiological characteristics, it is worth highlighting the higher reporting of linezolid sensitivity when compared to daptomycin (80.23% vs 72.24%) which has been similarly observed in other studies [7]. This observation will certainly add to the ongoing debate of the preferred presumptive alternative options for VRE BSIs [29]. Moreover, although many studies and guidelines advocate empirical addition of the aminoglycoside such as gentamycin for enterococcus BSIs in critically ill patients as a synergetic additive option, we report a high prevalence of gentamycin resistance amongst our cohort which suggests the prudence approach of using synergy testing beforehand or limiting it for difficult cases such as accompanied endocarditis [30].

While we acknowledge the retrospective and observational nature of the study which carries all associated limitations, we emphasize presented study is the largest from the region which spans a decade recoding the epidemiology of the disease and analyzing microbiological and clinical characteristics of an important pathogen. Furthermore, although projected results might mainly reflect local trends and observations, it can certainly consider as a milestone study that will aid in understanding trends of invasive bacterial infections as well as antimicrobial resistance profiles in the region.

In conclusion, evaluated enterococcal BSIs from large scale secondary care settings from Qatar over 10 years period demonstrated *E. faecalis* and E. faecium are the main pathogens, common in patients with premorbid conditions such as chronic kidney disease, and cancer mainly associated with admission to critical care and acquired through invasive devices with low-level resistance to vancomycin. Mortality remains high despite appropriate initial antimicrobial therapy which can partially be explained by the older cohort, comorbidities, and exiting underlying pathology. Strategies for the management, control, and prevention of enterococcal BSI should take into consideration risk factors, microbiological characteristics, recognizing susceptible patients to minimize risks of acquisitions.

## Ethical approval

The study was approved by Hamad Medical Corporation's Institutional Review Board (MRC-01-18-165) which is in line with international standards.

## Sources of funding

No funding was received towards the research.

## Author contribution

Conceptualization: GAA, WG, FSH and HS. Methodology and study design: GAA, WG and ASO. Data curation: GAA, WG, AA, AMA, MH and MMBA. Formal analysis and interpretation: GAA, WG, ASO and JD. Resources: AA and MAA. Writing – original draft preparation: GAA, WG and ASO. All authors critically reviewed and approved the version submitted for publication.

## Registration of research studies

The study was registered in the Research Registry database (https://www.researchregistry.com/) with unique number: **researchregistry8031**.All methods were performed in accordance with the relevant guidelines and regulations by MRC and the declaration of Helsinki.

## Guarantor

Dr Wael Goravey.

## Consent

The study was approved by Hamad Medical Corporation's Institutional Review Board with a waiver of informed consent (MRC-01-18-165) as retrospective study.

## Availability of data and materials

The datasets used and/or analyzed during the current study are available from the corresponding author on reasonable request.

## Provenance and peer review

Not commissioned, externally peer reviewed.

## Declaration of competing interest

The authors declare no conflict of interest concerning this manuscript.
